# Automatic Facial Expression Recognition in Standardized and Non-standardized Emotional Expressions

**DOI:** 10.3389/fpsyg.2021.627561

**Published:** 2021-05-05

**Authors:** Theresa Küntzler, T. Tim A. Höfling, Georg W. Alpers

**Affiliations:** ^1^Department of Politics and Public Administration, Center for Image Analysis in the Social Sciences, Graduate School of Decision Science, University of Konstanz, Konstanz, Germany; ^2^Department of Psychology, School of Social Sciences, University of Mannheim, Mannheim, Germany

**Keywords:** recognition of emotional facial expressions, software evaluation, human emotion recognition, standardized inventories, naturalistic expressions, automatic facial coding, facial expression recognition, specific emotions

## Abstract

Emotional facial expressions can inform researchers about an individual's emotional state. Recent technological advances open up new avenues to automatic Facial Expression Recognition (FER). Based on machine learning, such technology can tremendously increase the amount of processed data. FER is now easily accessible and has been validated for the classification of standardized prototypical facial expressions. However, applicability to more naturalistic facial expressions still remains uncertain. Hence, we test and compare performance of three different FER systems (Azure Face API, Microsoft; Face++, Megvii Technology; FaceReader, Noldus Information Technology) with human emotion recognition (A) for standardized posed facial expressions (from prototypical inventories) and (B) for non-standardized acted facial expressions (extracted from emotional movie scenes). For the standardized images, all three systems classify basic emotions accurately (FaceReader is most accurate) and they are mostly on par with human raters. For the non-standardized stimuli, performance drops remarkably for all three systems, but Azure still performs similarly to humans. In addition, all systems and humans alike tend to misclassify some of the non-standardized emotional facial expressions as neutral. In sum, emotion recognition by automated facial expression recognition can be an attractive alternative to human emotion recognition for standardized and non-standardized emotional facial expressions. However, we also found limitations in accuracy for specific facial expressions; clearly there is need for thorough empirical evaluation to guide future developments in computer vision of emotional facial expressions.

## 1. Introduction

Detecting emotional processes in humans is important in many research fields such as psychology, affective neuroscience, or political science. Emotions influence information processing (e.g., Marcus et al., 2000; Meffert et al., 2006; Fraser et al., 2012; Soroka and McAdams, 2015), attitude formation (e.g., Lerner and Keltner, 2000; Marcus, 2000; Brader, 2005), and decision making (Clore et al., 2001; Slovic et al., 2007; Pittig et al., 2014). One well-established strategy to measure emotional reactions of individuals is to track their facial expressions (Scherer and Ellgring, 2007; Keltner and Cordaro, 2017). The classic approach to analyse emotional facial responses is either an expert observation such as the Facial Action Coding System (FACS) (Sullivan and Masters, 1988; Ekman and Rosenberg, 1997; Cohn et al., 2007) or direct measurement of facial muscle activity with electromyography (EMG) (Cohn et al., 2007). Both are, however, time-consuming with respect to both, application and analysis.

A potential alternative to facilitate, standardize, and scale research on facial expressions is automatic image-based Facial Expression Recognition (FER), which has recently emerged from computer vision technology. Using machine learning, algorithms are being developed that extract emotion scores from observed facial expressions (Goodfellow et al., 2015; Arriaga et al., 2017; Quinn et al., 2017), which is considerably more time and cost efficient compared to classical approaches (Bartlett et al., 1999). FER is easily accessible to researchers of all fields and is increasingly used by the scientific community. Applications can be found, for example, in psychology, where such algorithms are used to predict mental health from social media images (Yazdavar et al., 2020), to validate interventions for autism (Wu et al., 2019), or to screen for Parkinson's disease (Jin et al., 2020). A sociological example is the assessment of collective happiness in society from social media images (Abdullah et al., 2015). In political science, one example is the study of representation of politicians in the media using FER (Boxell, 2018; Peng, 2018; Haim and Jungblut, 2020). Furthermore, the technology is used in consumer and market research, for example to predict advertisement efficiency (Lewinski et al., 2014; Teixeira et al., 2014; Bartkiene et al., 2019).

### 1.1. Prototypical vs. Naturalistic Facial Expressions

Training and testing of FER tools is typically conducted on data sets, which contain prototypical and potentially exaggerated expressions (Dhall et al., 2012). The images of these inventories are created under standardized (detailed instructions for the actors) and well-controlled conditions (e.g., lighting, frontal face angle; Lewinski et al., 2014; Calvo et al., 2018; Stöckli et al., 2018; Beringer et al., 2019; Skiendziel et al., 2019). As a result, the classification performance of FER systems and its generalizability to non-standardized and more naturalistic facial expressions is uncertain.

For prototypical facial expressions, FER also corresponds well to human FACS coding (Bartlett et al., 1999; Tian et al., 2001; Skiendziel et al., 2019) and non-expert human classification (Bartlett et al., 1999; Lewinski, 2015; Calvo et al., 2018; Stöckli et al., 2018). Accuracy is high for static images (Lewinski et al., 2014; Lewinski, 2015; Stöckli et al., 2018; Beringer et al., 2019) as well as for dynamic facial expressions from standardized inventories (Mavadati et al., 2013; Zhang et al., 2014; Yitzhak et al., 2017; Calvo et al., 2018). There is also growing evidence that FER provides valid measures for most emotion categories if naive participants are instructed to pose intense emotional facial expressions in a typical lab setting with frontal face recording and good lighting condition (Stöckli et al., 2018; Beringer et al., 2019; Sato et al., 2019; Kulke et al., 2020). However, all of these studies present their participants prototypical facial expression and instruct them to mimic these visual cues. This might result in an overestimation of FER performance in comparison to non-standardized facial expressions and moreover truly naturalistic emotional facial expressions.

Previous research also documents systematic misclassification of different FER systems and emotion categories. For fear, studies find a consistently lower accuracy compared to other emotion categories (Lewinski et al., 2014; Stöckli et al., 2018; Skiendziel et al., 2019). Some studies also report a substantial decrease in accuracy for anger (Lewinski et al., 2014; Stöckli et al., 2018; Dupré et al., 2020), whereas Skiendziel et al. (2019) report an improvement of this measurement in their study. Less consistently, sadness (Lewinski et al., 2014; Skiendziel et al., 2019) and disgust are also found to be error prone (Skiendziel et al., 2019). In contrast, the facial expression of joy is systematically classified with the highest accuracy (Stöckli et al., 2018; Skiendziel et al., 2019; Dupré et al., 2020). When looking at confusion between emotions in prior studies, FaceReader shows a tendency toward increased neutral measures for all other emotions (Lewinski et al., 2014) and a tendency to misclassify fearful faces as surprise (Stöckli et al., 2018; Skiendziel et al., 2019). Studies that compared different FER systems consistently find a large variation in performance between systems (Stöckli et al., 2018; Dupré et al., 2020) which underlines the need for comparatives studies.

Besides a general lack of studies, that directly compare different FER systems, empirical validation of FER to recognize emotional facial expressions is limited to intensely posed expressions. In contrast to those images, naturalistic or spontaneous facial expressions show stronger variations and are often less intense in comparison to standardized facial expressions (Calvo and Nummenmaa, 2016; Barrett et al., 2019). For example Sato et al. (2019) find a strong decrease in FER performance if participants respond spontaneously to imagined emotional episodes. Höfling et al. (2020) report strong correlations of FER parameters and participants' emotion ratings that spontaneously respond to pleasant emotional scenes, but find no evidence for a valid FER detection of spontaneous unpleasant facial reactions. Other studies report a decrease in FER emotion recognition for more subtle and naturalistic facial expressions (Höfling et al., 2021) and find a superiority of humans to decode such emotional facial responses (Yitzhak et al., 2017; Dupré et al., 2020). However, the data sets applied are still comprised of images collected in a controlled lab setting, with little variation on lighting, camera angle, or age of the subject which might further decrease FER performance under less restricted recording conditions.

### 1.2. Aims, Overview, and Expectations

In summary, FER offers several advantages in terms of efficiency and we already know that it performs well on standardized, prototypical emotional facial expressions. Despite many advantages of FER application and their validity to decode prototypical facial expression, the quality of the expression measurement and its generalizability to less standardized facial expressions is uncertain. Because the underlying algorithms remain unclear to the research community, including the applied machine-learning and its specific training procedure, empirical performance evaluation is urgently needed. Hence, this paper has two main aims: First, we provide an evaluation and a comparison of three widely used systems that are trained to recognize emotional facial expressions (FaceReader, Face++, and the Azure Face API) and compare them with human emotion recognition data as a benchmark. Second, we evaluate the systems on acted standardized and non-standardized emotional facial expressions: The standardized facial expressions are a collection of four facial expression inventories created in a lab setting displaying intense prototypical facial expressions [The Karolinska Directed Emotional Faces (Lundqvist et al., 1998), the Radboud Faces Database (Langer et al., 2010), the Amsterdam Dynamic Facial Expression Set (Van der Schalk et al., 2011), and the Warsaw Set of Emotional Facial Expression (Olszanowski et al., 2015)]. To approximate more naturalistic emotional expressions, we use a data set of non-standardized facial expressions: The Static Facial Expressions in the Wild data set (Dhall et al., 2018), which is built from movie scenes and covers a larger variety of facial expressions, lighting, camera position, and actor ages.

FER systems provide estimations for the intensity of specific emotional facial expressions through two subsequent steps: The first step is face detection including facial feature detection and the second step is face classification into an emotion category. For face detection, we expect that different camera angles, but also characteristics of the face such as glasses or beards will increase FER face detection failures resulting in higher rates of drop out. We expect the standardized expressions to result in less drop out due to failures in face detection, since the camera angle is constantly frontal, and no other objects such as glasses obstruct the faces. Correspondingly, we expect more drop out in the non-standardized data set, which means there are more images where faces are not detected, since the variability of the facial expressions is higher. For the second step (i.e., emotion classification), we expect strong variation between emotion categories (e.g., increased performance for joy faces, decreased performance on fear faces). We further expect a tendency toward the neutral category and a misclassification of fear as surprise. As explained for the drop outs, we assume the non-standardized images to be more variable and therefore more difficult to classify. The overall performance on the non-standardized data is therefore expected to be lower. This research provides important information about the generalizability of FER to more naturalistic, non-standardized emotional facial expressions and moreover the performance comparison of specific FER systems.

## 2. Materials and Methods

We use three different facial expression recognition tools and human emotion recognition data to analyze emotional facial expressions in more or less standardized facial expressions. As an approximation to standardized and non-standardized facial expressions we analyze static image inventories of actors who were instructed to display prototypical emotional expressions and, in addition, an inventory of actors displaying more naturalistic emotional facial expressions in movie stills. We extract probability parameters for facial expressions corresponding to six basic emotions (i.e., joy, anger, sadness, disgust, fear, surprise, and neutral) from all tools. As a benchmark, we collect data from human raters who rated subsets of the same images.

### 2.1. Images of Facial Expressions

We test the different FER tools as well as human facial recognition data on standardized and non-standardized emotional facial expressions displayed in still images. All selected inventories are publicly available for research and contain emotional facial expression images of the basic emotion categories. Table 1 displays the emotion categories and image distributions for both data sets (i.e., standardized and non-standardized) including drop out rates specifically for the three FER tools.

**Table 1:** Category distributions of test data and drop outs of Azure, Face++, and FaceReader.

	**Standardized data**							
	**Neutral**	**Happy**	**Sad**	**Fear**	**Angry**	**Surprise**	**Disgust**	**Overall**
Absolute frequency of images	178	178	178	178	178	178	178	1246
Relative frequency of images	14.3	14.3	14.3	14.3	14.3	14.2	14.2	100
	**Drop out rates (percent per category)**							
Face++	0.0	0.0	0.0	0.0	0.0	0.0	0.0	0.0
Azure	0.0	0.0	0.0	0.0	0.0	0.0	0.0	0.0
FaceReader	0.6	0.0	0.0	1.1	1.1	2.2	1.2	0.88
	**Non-standardized data**							
	**Neutral**	**Happy**	**Sad**	**Fear**	**Angry**	**Surprise**	**Disgust**	**Overall**
Absolute frequency of images	236	270	245	143	254	151	88	1387
Relative frequency of images	17.0	19.5	17.7	10.3	18.3	10.9	6.3	100
	**Drop out rates (percent per category)**							
Face++	0.0	0.7	0.8	0.7	0.4	0.0	1.1	0.5
Azure	16.9	11.1	25.3	26.6	25.2	17.9	23.9	20.3
FaceReader	73.3	70.0	75.1	79.0	76.8	74.8	69.3	74.2

*Percentages are rounded to the first decimal. The base of the percentage is the respective total of each category. Reading example: Azure did not find a face in 16.9% of the 236 neutral images of the non-standardized data and a total of 20.3% of the 1387 images dropped out because of no face detection*.

Standardized facial expressions are a collection of images created in the lab with controlled conditions (i.e., good lighting, frontal head positions, directed view) displaying prototypical expressions of clearly defined emotions. In order to maximize image quantity and introduce more variability, the prototypical images consist of four databases: (1) The Karolinska Directed Emotional Faces contains images of 35 males and 35 females between 20 and 30 years old (Lundqvist et al., 1998). The present study uses all frontal images (resolution: 562 × 762). (2) The Radboud Faces Database, which contains images of facial expressions of 20 male and 19 female Caucasian Dutch adults (Langer et al., 2010). We used the subset of adult models looking straight into the camera with images taken frontal (resolution: 681 × 1,024). (3) The Amsterdam Dynamic Facial Expression Set, from which we used the still image set (resolution: 720 × 576). The models are distinguished between being Northern-European (12 models, 5 females) and Mediterranean (10 models, 5 of them female; Van der Schalk et al., 2011). (4) The Warsaw Set of Emotional Facial Expression offers images of 40 models (16 females, 14 males) displaying emotional facial expressions (Olszanowski et al., 2015). Images are taken frontal and the complete set is used in this study (resolution: 1,725 × 1,168). This results in an overall of 1,246 images evenly distributed over the relevant emotion categories.

Non-standardized facial expressions stem from a data set that was developed as a benchmark test for computer vision research for more naturalistic settings. The Static Facial Expressions in the Wild (SFEW) data set consists of stills from movie scenes that display emotions in the actors' faces. Examples of movies are “Harry Potter” or “Hangover” (Dhall et al., 2018). This study uses the updated version (Dhall et al., 2018). The data set was compiled using the subtitles for the deaf and hearing impaired and closed caption subtitles. These subtitles contain not only the spoken text, but additional information about surrounding sounds, such as laughter. The subtitles were automatically searched for words suggesting emotional content. Scenes resulting from this search were then suggested to trained human coders, who classified and validated the final selection of emotional facial expressions for this inventory (Dhall et al., 2012). We use these images to rigorously test how well the systems perform on images that are not prototypical and not taken under standardized conditions (variable lighting and head positions). The inventory consists of 1,387 images (resolution: 720 × 576) which are unevenly distributed across emotion categories (minimum of 88 images for disgust and a maximum of 270 images for joy).

### 2.2. Facial Expression Recognition Tools

We test three FER tools: The Microsoft Azure Face API (Version 1.0, Microsoft), Face++ (Version 3.0, Megvii Technology) and FaceReader (Version 8.0, Noldus Information Technology). The first two are easily accessible APIs, which also offer a free subscription. FaceReader is a software to be installed locally on a computer and is well-established in the research community. Each of the systems allow to analyse faces in images, with functions such as face detection, face verification, and emotion recognition. They all provide probability scores for neutral, joy, sadness, anger, disgust, fear, and surprise. While scores of Azure and FaceReader are between 0 and 1, Face++ uses a scale from 1 to 100. We thus rescale Face++ scores to 0 to 1. FaceReader specifically provides an additional quality parameter and it is suggested to remove images, if the quality of face detection is too low. Therefore, we remove all images with a quality parameter below 70%.

### 2.3. Human Emotion Recognition

As a benchmark for the FER results we collected emotion recognition data of humans who each rate a random subsample of up to 127 of the 2,633 images each in an online study. Participants who rated less than 20 images are excluded for further analyses (17 participants rated between 20 and 126 pictures). This results in 101 participants (58 female, 42 male, 1 diverse, *M*_*age*_ = 29.2, *SD*_*age*_ = 9.1) who rated on average 116.1 (SD = 28.1) images. Twenty-five images were randomly not rated by any participants (<1%). Participants were instructed to classify facial expression as neutral, joy, sadness, anger, disgust, fear, surprise, or another emotion. Multiple choices were possible. In addition, the perceived genuineness of the expressed emotion was rated on a 7-point Likert scale (1 -very in-genuine, 7 -very genuine). All ratings are averaged per image to improve comparability to the metric provided by the FER tools. This results in percentages of emotion ratings and average values per image for the genuineness ratings.

### 2.4. Analyses

First, we analyze the human raters' scores for perceived genuineness and emotion classification as a manipulation check for the two data sets of facial expressions. Differences between the genuineness of non-standardized vs. standardized facial expressions are tested statistically for all images as well as separately for all emotion categories utilizing independent *t*-tests. Correspondingly, we analyze the human emotion recognition data to provide a benchmark for the FER comparison. Again we statistically test for differences between non-standardized vs. standardized facial expressions for all emotion categories utilizing independent *t*-tests. In addition, we calculate one-sample *t*-tests against zero to estimate patterns of misclassification within human emotion recognition. Cohen's d is reported for all *t*-tests.

Second, we test the performance of face detection. As described above, FER is a two step process of first face detection and second emotion classification. To test performances on face detection, we check for how many images a specific tool gives no result (drop out rate).

Third, we calculate several indices of emotion classification (i.e., accuracy, sensitivity, and precision) for the three FER tools to report performance differences descriptively. In order to evaluate emotion classification, each algorithm's output is compared to the original coding of the intended emotional facial expression category (i.e., ground truth). The different tools return values for each emotion category. We define the category with the highest certainty as the chosen one, corresponding to a winner–takes–all principle^1^. A general indication of FER performance is the accuracy, which is the share of correctly identified images out of all images, where a face is processed (thus, excluding drop out)^2^. Other excellent measures to evaluate emotion classification are category specific sensitivity and precision. Sensitivity describes the share of correctly predicted images out of all images truly in the respective category. It is a measure of how well the tool does in detecting a certain category. Precision is the share of correctly predicted images out of all images predicted as one category. In other words, precision is a measure of how much we can trust the categorization of the tool. In order to identify patterns of classifications, we additionally build confusion matrices for the FER measurement and true categories.

Fourth, we report differences in emotion recognition performance between the three systems and human data with Receiver Operating Characteristic (ROC) analysis and statistical testing of the corresponding Area Under the Curve (AUC). ROC analysis is initially a two-class classification strategy. In order to apply the ROC rationale to a multi-class classification, we consider each probability given to a category as one observation. In other words, each image makes up for seven observations for each tool. The ROC curve plots a true positive share against a false positive share for varying probability thresholds above which a category is considered correct. A good classifier gives low probabilities to wrong classifications and high probabilities to correct classifications. This is measured by the AUC. Better classifiers give larger AUCs. We compare AUCs of the different algorithms pairwise, using a bootstrapping method with 2,000 draws (Robin et al., 2011).

Analyses are conducted in R (R Core Team, 2019), using the following packages (alphabetical order): caret (Kuhn, 2020), data.table (Dowle and Srinivasan, 2020), dplyr (Wickham et al., 2020), extrafont (Chang, 2014), ggplot2 (Wickham, 2016), httr (Wickham, 2020), jsonlite (Ooms, 2014), patchwork (Pedersen, 2020), plotROC (Sachs, 2017), pROC (Robin et al., 2011), purrr (Henry and Wickham, 2020), RColorBrewer (Neuwirth, 2014), stringr (Wickham, 2019), tidyverse (Wickham et al., 2019).

## 3. Results

### 3.1. Human Raters: Genuineness of Facial Expressions

**Figure 1:**
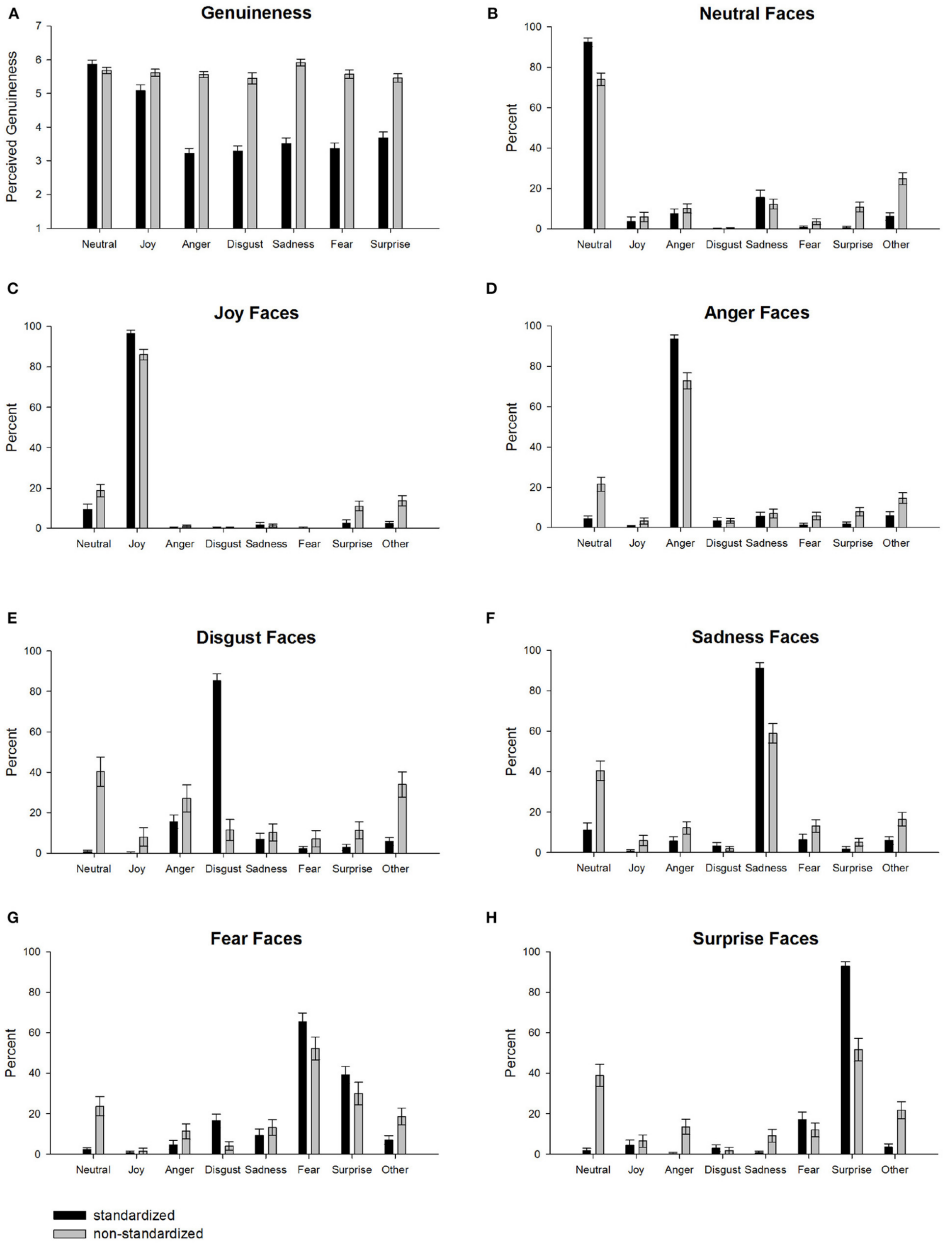
Averaged human ratings separately for basic emotion categories for standardized (black bars) and non-standardized facial expressions (gray bars). **(A)** Depicts mean genuineness ratings ranging from 1 (very in-genuine) to 7 (very genuine). **(B–H)** Depict mean emotion ratings (percent) for **(B)** neutral, **(C)** joy, **(D)** anger, **(E)** disgust, **(F)** sadness, **(G)** fear, and **(H)** surprise expressions. Error bars are 95% confidence intervals.

We test for differences between standardized and non-standardized facial expression inventories regarding their perceived genuineness (see Figure 1A). Analysis shows that the non-standardized facial expressions are perceived as much more genuine compared to the standardized facial expressions [standardized inventories: *M* = 4.00, *SD* = 1.43; non-standardized inventory: *M* = 5.64, *SD* = 0.79; *t*(2606) = 36.58, *p* < 0.001, *d* = 1.44]. In particular, non-standardized facial expressions are rated as more genuine for anger, *t*(426) = 27.97, *p* < 0.001, *d* = 2.75, sadness, *t*(418) = 25.55, *p* < 0.001, *d* = 2.43, fear, *t*(317) = 21.10, *p* < 0.001, *d* = 2.38, disgust, *t*(263) = 18.10, *p* < 0.001, *d* = 2.36, surprise, *t*(322) = 16.02, *p* < 0.001, *d* = 1.79, and joy, *t*(441) = 5.58, *p* < 0.001, *d* = 0.54, whereas among the standardized inventories neutral facial expressions are rated more genuine, *t*(407) = 2.36, *p* = 0.019, *d* = 0.24. These results support the validity of the selection of image test data—the standardized facial expressions are perceived less genuine compared to the non-standardized facial expressions.

### 3.2. Human Raters: Emotion Recognition

Next, we analyze the human emotion ratings (see Figures 1B–H). Comparisons against zero show that for most emotion categories, classifications are highest for the correct category. The only exception are non-standardized disgust faces that are more often categorized as angry, *t*(87) = 7.99, *p* < 0.001, *d* = 0.85, than disgusted, *t*(87) = 4.40, *p* < 0.001, *d* = 0.47. In addition, fearful faces are also misclassified (or at least co-classified) as surprise for standardized, *t*(175) = 18.22, *p* < 0.001, *d* = 1.37, and non-standardized facial expressions, *t*(142) = 10.69, *p* < 0.001, *d* = 0.89. A comparison between standardized and non-standardized data reveals a strong increase in neutral ratings for non-standardized emotion categories [disgust: *t*(263) = 15.03, *p* < 0.001, *d* = 1.96; surprise: *t*(322) = 14.33, *p* < 0.001, *d* = 1.60; fear: *t*(317) = 9.54, *p* < 0.001, *d* = 1.07; sadness: *t*(418) = 9.01, *p* < 0.001, *d* = 0.89; anger: *t*(426) = 7.96, *p* < 0.001, *d* = 0.78; joy: *t*(441) = 4.26, *p* < 0.001, *d* = 0.41]. Correspondingly, non-standardized facial expressions show a strong decrease in the correct emotion category compared to standardized facial expressions for some categories [disgust: *t*(263) = 24.63, *p* < 0.001, *d* = 3.21; surprise: *t*(322) = 14.35, *p* < 0.001, *d* = 1.60; sadness: *t*(418) = 10.28, *p* < 0.001, *d* = 1.02; neutral: *t*(407) = 8.99, *p* < 0.001, *d* = 0.90; anger: *t*(426) = 8.03, *p* < 0.001, *d* = 0.79; joy: *t*(441) = 5.83, *p* < 0.001, *d* = 0.57; fear: *t*(317) = 3.79, *p* < 0.001, *d* = 0.43]. Taken together, non-standardized compared to standardized facial expressions are perceived more often as neutral and less emotionally intense on average.

### 3.3. FER Systems: Drop Out

To evaluate the step of face detection, we report drop out rates separately for each FER tool in Table 1. Drop out for the standardized data is nearly non-existent, however, strong differences can be reported for the non-standardized data set. Azure returns no face detection for around 20% of the images. For FaceReader, the drop out is even higher with 74%^3^. This result partially confirms our expectations, as for Azure and FaceReader the drop out in the non-standardized data is much higher than among the standardized data. In contrast, Face++ shows superior face detection with nearly no drop out for the non-standardized data. See Supplementary Table 1 for statistical comparison of the drop out rates.

### 3.4. FER Systems: Emotion Recognition

To descriptively compare classification performance, we report accuracies for each tool on each data set, along with category specific sensitivity and precision (Table 2). Details on the statistical comparisons can be found in Supplementary Table 2^4^. As expected, accuracy is better for all tools on the standardized data. FaceReader performs best, with 97% of the images classified correctly. The difference to both Azure and Face++ is significant (*p* < 0.001). Azure and Face++ perform similarly, *p* = 0.148, both put around 80% of the images in the correct category. For the non-standardized data, accuracy is much lower. Azure performs best, still correctly classifying 56% of the images. FaceReader and Face++ both correctly classify only about one third of the non-standardized images which constitutes a significant decrease of accuracy compared to Azure (*p* < 0.001).

**Table 2:** Sensitivity, precision, and accuracy of Azure, Face++, and FaceReader separately for emotion categories.

	**Azure**				**Face++**				**FaceReader**			
	**Stand**.		**Non-Stand**.		**Stand**.		**Non-Stand**.		**Stand**.		**Non-Stand**.	
	**Sens**	**Prec**	**Sens**	**Prec**	**Sens**	**Prec**	**Sens**	**Prec**	**Sens**	**Prec**	**Sens**	**Prec**
Neutral	1.00	0.63	0.94	0.38	0.94	0.70	0.40	0.34	0.99	0.92	0.68	0.2
Joy	1.00	0.98	0.85	0.88	0.99	0.96	0.48	0.76	1.00	0.99	0.42	0.92
Anger	0.51	0.91	0.38	0.87	0.49	0.84	0.15	0.36	0.96	0.99	0.14	0.42
Disgust	0.85	0.98	0.10	0.50	0.89	0.77	0.16	0.17	0.97	0.99	0.15	0.17
Sadness	0.88	0.75	0.48	0.77	0.81	0.75	0.19	0.40	0.98	0.97	0.16	0.32
Fear	0.46	0.99	0.03	0.33	0.40	0.95	0.18	0.18	0.88	0.97	0.00	0.00
Surprise	0.98	0.73	0.56	0.43	0.97	0.71	0.66	0.20	0.98	0.93	0.34	0.33
Average	0.81	0.85	0.48	0.59	0.79	0.81	0.32	0.35	0.97	0.97	0.27	0.34
Accuracy	0.81		0.57		0.79		0.32		0.97		0.31	

*Stand., standardized data; Non-Stand., non-standardized data; Sens., sensitivity; Prec., precision*.

Looking at the specific emotion categories and their performance indices, joy expressions are classified best. For the standardized data, both sensitivity and precision are or nearly are all 1. Also for the non-standardized data, the joy category is classified best. However, Azure is the only software with overall acceptable performance. In the standardized angry category, all tools show high precision, however Azure and Face++ lack in sensitivity. For the non-standardized angry category, only Azure's precision is acceptable. Face++, and FaceReader do not perform reliably. Performance on the other categories on the standardized data resembles each other: FaceReader clearly outperforms the other tools. In contrast, for the non-standardized facial expressions, Azure performs best, although the values are substantially decreased in comparison to standardized facial expressions.

**Figure 2:**
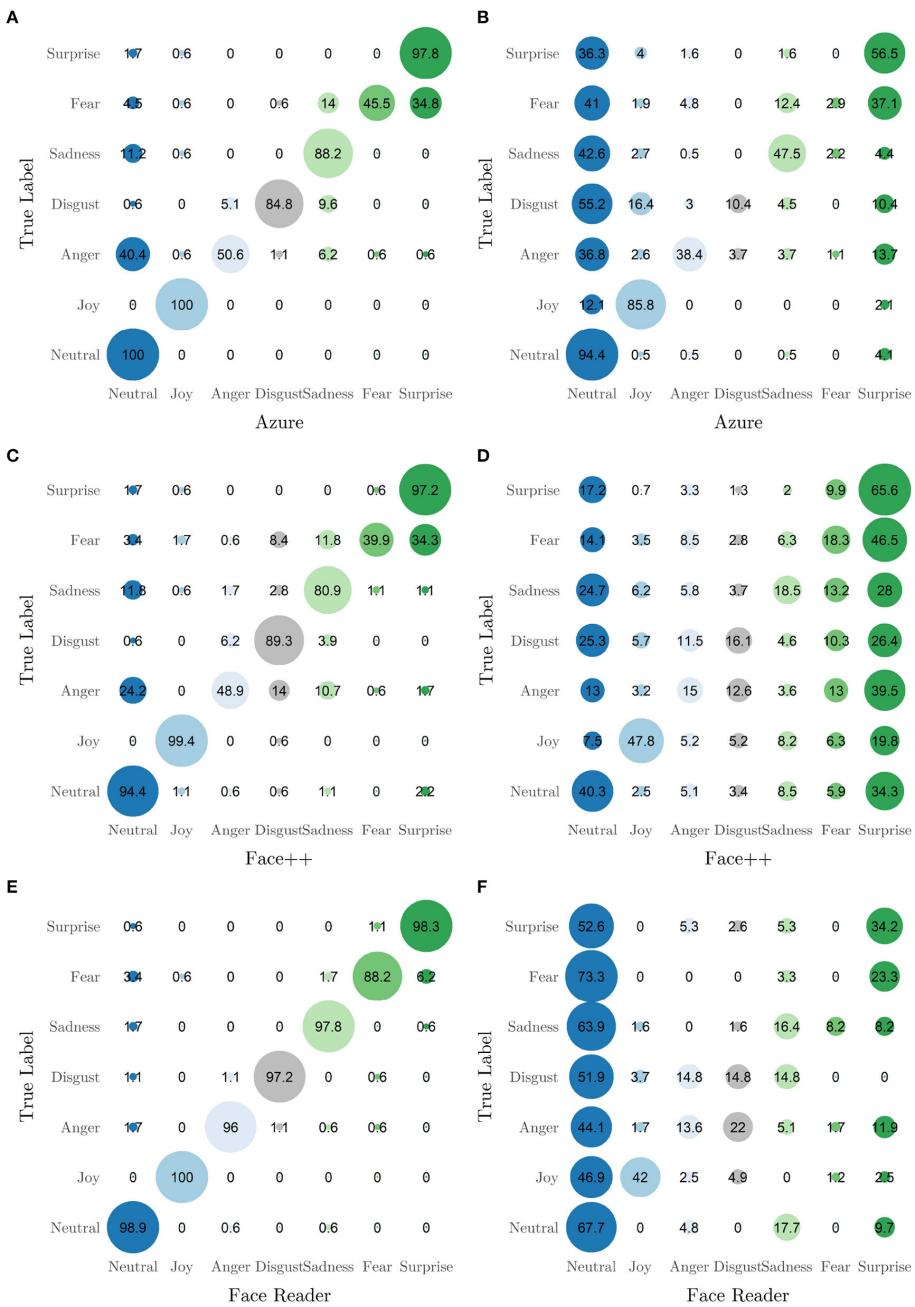
Confusion matrices indicating classification performance on standardized (left panels) and non-standaridzed data (right panels): **(A)** standardized data by Azure, **(B)** non-standardized data by Azure, **(C)** standardized data by Face++, **(D)** non-standardized data by Face++, **(E)** standardized data by FaceReader and **(F)** non-standardized data by FaceReader. Numbers indicate percentages to the base of the true category. Reading example: From the standardized data Azure classifies 4.5% of the truly fearful expressions as neutral. The 45.5% of the fearful images are classified correctly.

To study confusion rates between categories, Figure 2 depicts confusion matrices between the true labels and the highest rated emotion by each software. In the standardized data, all three tools show the pattern of classifying fearful expressions as surprise or sadness. The confusion between fear and surprise is expected, whereas the confusion of fear with sadness is new. Additionally, Azure and Face++ show a tendency to misclassify anger, sadness and fear as neutral. For FaceReader, this tendency is observable to a smaller extent. This reflects partially the expected tendency toward a neutral expression. In the non-standardized data set, all applications show a pronounced tendency toward the neutral category. Additionally, Face++ shows a trend toward surprise, sadness and fear. To a smaller extend, the misclassification to surprise and sadness is problematic in Azure and FaceReader alike.

### 3.5. Humans vs. FER: Comparison of Emotion Recognition

**Figure 3:**
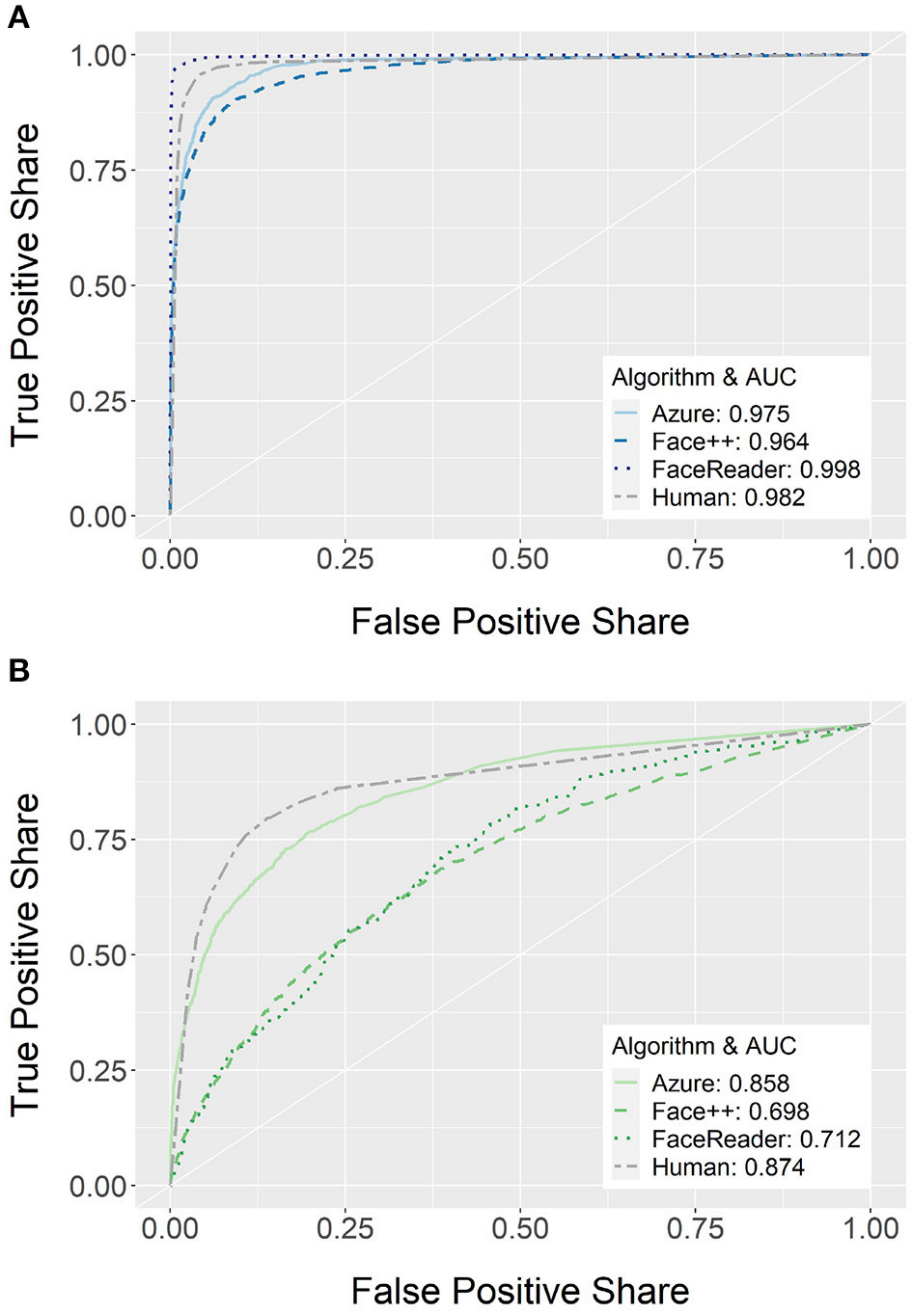
Classification performance depicted as Receiver Operating Characteristic (ROC) curves and corresponding Area under the Curve (AUC) for overall emotion recognition performance for the three FER systems (Azure, Face++, and FaceReader) and human raters. Separately for **(A)** standardized facial expressions and **(B)** non-standardized facial expressions separately. The white diagonal line indicates classification performance by chance.

To directly compare all sources of emotion recognition, we calculate ROC curves and report them in Figure 3 along with the corresponding AUCs. ROC curves for specific emotion categories are shown in Supplementary Figure 2 and corresponding statistical comparisons are reported in Supplementary Table 5^5^.

For the standardized facial expressions (see Figure 3A), humans, overall, recognize them significantly better than Azure, *p* = 0.035, and Face++, *p* < 0.001. However, FaceReader performs significantly better than humans on such facial expressions, *p* < 0.001. While the same pattern holds true for fear faces (Azure: *p* = 0.003; FaceReader: *p* < 0.001, Face++: *p* < 0.001), all algorithms perform significantly better than humans for neutral (Azure: *p* < 0.001; FaceReader: *p* < 0.001, Face++: *p* < 0.001), joy (Azure: *p* = 0.023; FaceReader: *p* = 0.024, Face++: *p* = 0.027), and surprise expressions (Azure: *p* = 0.012; FaceReader: *p* = 0.012, Face++: *p* = 0.013). Also, for standardized facial expressions of disgust, FaceReader, *p* = 0.002, and Face++, *p* = 0.023, perform better compared to humans while Azure is comparable to humans, *p* = 0.450. Regarding anger, FaceReader, and humans perform comparably, *p* = 0.353, and both outperform Azure and Face++, *p* < 0.001. Finally, FaceReader shows better classification of sad faces compared to Azure, *p* = 0.078, Face++, *p* < 0.001, and humans, *p* = 0.021.

For the non-standardized facial expressions (see Figure 3B), humans overall show similar performance to Azure, *p* = 0.058, and both perform better than FaceReader, *p* < 0.001, and Face++, *p* < 0.001. While this pattern is the same for joy (Azure: *p* = 0.554; FaceReader: *p* < 0.001, Face++: *p* < 0.001) and sadness (Azure: *p* = 0.448; FaceReader: *p* < 0.001, Face++: *p* < 0.001), humans outperform all algorithms in the detection of anger (Azure: *p* < 0.001; FaceReader: *p* < 0.001, Face++: *p* < 0.001) and fear facial expressions (Azure: *p* < 0.001; FaceReader: *p* < 0.001, Face++: *p* < 0.001). In contrast, Azure performs better than humans regarding neutral, *p* < 0.001, and disgust faces, *p* < 0.001, while FaceReader (neutral: *p* < 0.001; disgust: *p* = 0.002) and Face++ (neutral: *p* = 0.001; disgust: *p* = 0.023) show equal or worse performance compared to humans. Finally, Azure, *p* = 0.006, and Face++, *p* < 0.001, performs better than humans in the detection of non-standardized surprise facial expressions where FaceReader performs similar to humans, *p* = 0.535.

Taken together, for most emotion categories there is at least one FER system that performs equally well or better compared to humans. The only exceptions are non-standardized expressions of fear and anger, where humans clearly outperform all FER systems. FaceReader shows particularly good performance for standardized facial expressions and Azure performs better on non-standardized facial expressions.

## 4. Discussion

In this paper, we evaluate and compare three widely used FER systems, namely Azure, Face++ and FaceReader, and human emotion recognition data. For the performance comparison, we use two different kinds of emotional facial expression data sets: First, a standardized data set comprised of lab generated images displaying intense, prototypical facial expressions of emotions under very good recording conditions (i.e., lighting, camera angle). Second, we test a non-standardized set, which contains facial expressions from movie scenes depicting emotional faces as an approximation for more naturalistic, spontaneous facial expressions (Dhall et al., 2018). The non-standardized facial expressions constitute an especially difficult test case, since it contains large variation in the expressions itself, the surrounding circumstances and the displayed person's characteristics.

Overall, the three classifiers as well as humans perform well on standardized facial expressions. However, we observe large variation and a general decrease in performance for the non-standardized data, in line with previous work (Yitzhak et al., 2017; Dupré et al., 2020). Although emotion recognition performance is generally lower for such facial expressions, FER tools perform similarly or better than humans for most emotion categories of non-standardized (except for anger and fear) and standardized facial expressions. Facial expressions of joy are detected best among the emotion categories in both standardized and non-standardized facial expressions, which also replicates existing findings (Stöckli et al., 2018; Höfling et al., 2021). However, FER performance varies strongly between systems and emotion categories. Depending on the data and on which emotions one aims at classifying, one algorithm might be better suited than the other: Face++ shows almost no drop out in face detection even under the non-standardized condition, FaceReader shows excellent performance for standardized prototypical facial expressions and outperforms humans, and Azure shows superior overall performance on non-standardized facial expressions among all FER tools.

### 4.1. Implications for Application

From our data, we can derive three broad implications. First, all FER tools perform much better on the standardized, prototypical data, than on the non-standardized, more naturalistic data. This might indicate over fitting on standardized data. Second, FER systems and human coders can detect some emotion categories better than others, resulting in asymmetries in classification performance between emotion categories. This indicates that the detection of certain emotional facial expressions is generally more error prone than others. Third, we can identify performance problems that are specific to FER tools.

First, as expected, all FER systems perform better on the standardized compared to non-standardized and more naturalistic facial expressions. This is the case for both face detection and emotion classification. Within the standardized data, face detection is near to prefect for all systems and shows almost no drop out based on face detection failures. Regarding the emotion classification, FaceReader outperforms Face++, Azure, and even human coders. Within the non-standardized data, face detection is observed to be problematic for Azure and FaceReader. Judging the classification performance on the non-standardized data set, all three classifiers show a large overall decrease in accuracy, whereby Azure is most accurate compared to Face++ and FaceReader. In particular, all FER systems, and less pronounced in humans, show a misclassification of emotional facial expressions as neutral facial expressions for the non-standardized data. This is an important observation not shown by Dupré et al. (2020), since they have not reported confusions with the neutral category. We suspect the neutral classification due to the expressions in acted films being less intense compared to standardized, lab generated data. Hence, the vastly better performance on standardized, prototypical facial expressions which were generated under controlled conditions may indicate limitations of FER systems to more naturalistic and more subtle emotional facial expressions.

Second, we observe that FER and human performance reflect varying underlying difficulties in the classification of different emotions. In other words, certain emotions are harder to detect than others, for example because of more subtle expressions or less distinct patterns. This evolves from shared classification error patterns between the three algorithms which corresponds to prior research on other algorithms and human recognition performance. In our data, joy is recognized best and fear is among the most difficult to classify which is in line with prior FER (Stöckli et al., 2018; Skiendziel et al., 2019; Dupré et al., 2020) and human emotion recognition research (Nummenmaa and Calvo, 2015; Calvo and Nummenmaa, 2016). Anger has been found to be difficult to classify in some studies (Stöckli et al., 2018; Dupré et al., 2020), but not in others (Skiendziel et al., 2019). With regards to our findings, angry faces can be classified with low sensitivity, but high precision. Sadness and disgust are reported to be difficult to detect in other studies (Lewinski et al., 2014; Skiendziel et al., 2019). Fear is regularly misclassified as surprise, as found in other studies with FER (Stöckli et al., 2018; Skiendziel et al., 2019) and humans alike (Palermo and Coltheart, 2004; Calvo and Lundqvist, 2008; Tottenham et al., 2009; Calvo et al., 2018). For the non-standardized data, FER performance on disgust is among the lowest for all classifiers which corresponds to human recognition data in the present study. In line with previous research, the pronounced performance drop for many non-standardized images compared to standardized emotion categories (Yitzhak et al., 2017; Dupré et al., 2020) might indicate that the FER systems are not trained on detecting the full variability of emotional facial expressions. Importantly, these results reflect that FER simulates human perception and also shows similar classification errors.

Third, we make a series of observations, that specific FER systems misclassify certain emotion categories, which is not shared by human coders. In our data, fear is also misclassified as sadness by Azure in standardized and non-standardized facial expressions. For the non-standardized data, we also report a general tendency to misclassify surprise expressions, that is not evident in other studies. Especially the misclassification toward surprise in the non-standardized data might be explained by an open mouth due to speaking in movies, for which the applications do not account. In addition, Face++ misclassifies any emotion in the non-standardized data as fear and to a lesser extend as sadness. Regarding FaceReader, we observe a pronounced misclassification of naturalistic facial expressions as neutral. These findings indicate misclassification pattern specific for the three investigated FER systems which possibly reflect differences in their machine-learning architecture, training material and validation procedure.

### 4.2. Limitations and Outlook

This study has some limitations. Most obviously, we compare three representative and not all available software systems on the market. While we choose software that is widely used, other algorithms will need to be examined in a similar fashion. For example, Beringer et al. (2019) find that FACET shows a certain resilience to changes in lighting and camera angle on lab generated data. We could not see in this study if this resilience transfers to an even harder task.

To approximate more naturalistic facial expressions, we utilize images from movie stills as the non-standardized data set. While this is convenient and emotional expressions are already classified and evaluated, these images are of course also posed by actors. However, good acting is generally thought of as a realistic portrayal of true affect. Our ratings of genuineness appear to support our distinction of standardized and non-standardized facial expressions. In addition, our human recognition data provide further validation of emotion categorization of this particular facial expression inventory. Even though acted portrays of emotional facial expressions differ between prototypical inventories and movies, which is in line with previous research (Carroll and Russell, 1997), these acted facial expressions are only approximations for true emotional expressions. Moreover, movie stimuli may be rated as more authentic compared to the prototypical data, due to many reasons like the variation in head orientations, lighting, backgrounds, and familiarity with the actors or movie plot. Hence, facial expressions of true emotion require an additional criterion of emotional responding like ratings of currently elicited emotions.

Furthermore, we argue that FER would be most useful in categorizing spontaneous and naturalistic facial expressions in different contexts. The SFEW data set serves as an approximation for this. However, it is unclear whether the displayed emotional facial expressions are grounded in emotional processing or just simulated. For example, Höfling et al. (2020) elicited spontaneous emotional responses by presenting emotional scenes to their participants and found FER detects changes in facial expressions only for pleasant emotional material. Hence, more data sets are needed to test different naturalistic settings and foster development in this area.

Beyond the bias in FER toward prototypical expressions under good condition, there are other sources of systemic error that we did not address, such as biases against race, gender, age, or culture (Zou and Schiebinger, 2018; Aggarwal et al., 2019; Wellner and Rothman, 2020). For example, it has been shown that automated facial analysis to classify gender works less well for people with a darker skin tone (Buolamwini and Gebru, 2018). Many training data sets are concentrated on Northern America and Europe (Shankar et al., 2017), which partially causes the biases and at the same time makes it difficult to detect them. Future research should take these variables into account to evaluate measurement fairness independent of specific person characteristics.

## 5. Conclusion

This study contributes to the literature by comparing the accuracy of three state-of-the-art FER systems to classify emotional facial expressions (i.e., FaceReader, Azure, Face++). We show that all systems and human coders perform well for standardized, prototypical facial expressions. When challenged with non-standardized images, used to approximate more naturalistic expressions collected outside of the lab, performance of all systems as well as human coders drops considerably. Reasons for this are substantial drop out rates and a decrease in classification accuracy specific to FER systems and emotion categories. With only a short history, FER is already a valid research tool for intense and prototypical emotional facial expressions. However, limitations are apparent in the detection of non-standardized facial expressions as they may be displayed in more naturalistic scenarios. Hence, further research is urgently needed to increase the potential of FER as a research tool for the classification of non-prototypical and more subtle facial expressions. While the technology is, thus, a promising candidate to assess emotional facial expressions on a non-contact basis, researchers are advised to interpret data from non-prototypical expressions in non-restrictive settings (e.g., strong head movement) carefully.

## Data Availability Statement

The raw data supporting the conclusions of this article will be made available by the authors, without undue reservation.

## Ethics Statement

Ethical review and approval was not required for the study on human participants in accordance with the local legislation and institutional requirements. The patients/participants provided their written informed consent to participate in this study.

## Author Contributions

TK conceived and designed the study, contributed Face++ and Azure data, conducted the analysis and interpretation of the results, and also drafted the work. TH contributed FaceReader data and collected data from human raters. TH and GA contributed to the interpretation of the results and writing of the manuscript. All authors contributed to the article and approved the submitted version.

## Conflict of Interest

The authors declare that the research was conducted in the absence of any commercial or financial relationships that could be construed as a potential conflict of interest.

## Footnotes

^1^We also test different thresholds, but there is no reasonable performance improvement to be gained (see Supplementary Figure 1).

^2^Since this procedure leads to different samples for each algorithm, especially among the non-standardized data, we also compute the analysis for the subsample of non-standard images, which are recognized by all algorithms. The results are reported in Supplementary Table 3. Differences are minor, qualitatively the results remain the same.

^3^Twenty percent of the images have a quality that is too low for FaceReader to reliably detect emotions and we therefore exclude these from the analysis, in 54% no face is found by FaceReader.

^4^Since drop out rates differ strongly between the algorithms, especially among the naturalistic data, we also compute the analysis for the subset of naturalistic images, which are recognized by all algorithms. Differences are minor with corresponding patterns (see also Supplementary Table 4). Additionally, we report the shares of correctly identified images based on all images in Supplementary Table 3.

^5^AUC analysis for the subset of non-standardized data passed by all algorithms yields the same results.
