# Supplementary material for: Automatic Facial Expression Recognition in Standardized and Non-standardized Emotional Expressions
Source: Front Psychol. 2021 May 5;12:627561. doi: 10.3389/fpsyg.2021.627561 (PMC8131548; doi:10.3389/fpsyg.2021.627561)
Supplement: Supplementary file 1 [file Data_Sheet_1.pdf]

Supplementary Material

1 SUPPLEMENTARY TABLES AND FIGURES

1.1 Figures

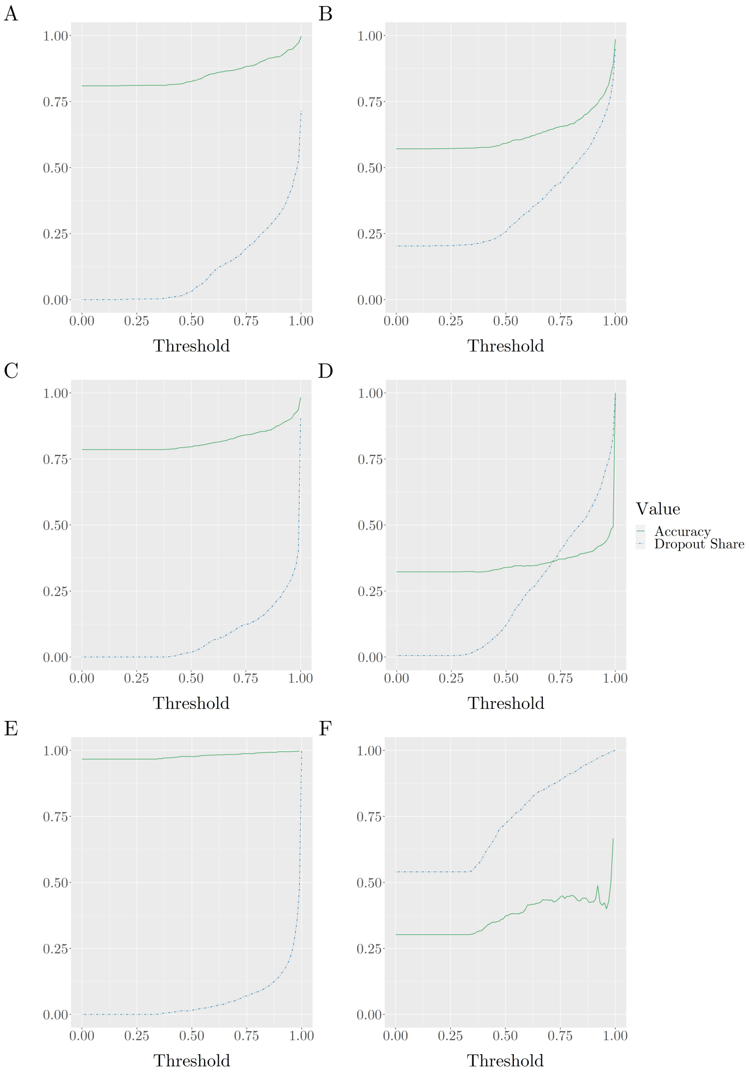

Figure S1. This Figure depicts the accuracy and drop out rate for varying thresholds for **(A)** the standardized data by Azure, **(B)** the non-standardized data by Azure, **(C)** the standardized data by Face++, **(D)** the non-standardized data by Face++, **(E)** the standardized data by FaceReader and **(F)** the non-standardized data by FaceReader. The threshold runs from 0 to 1 and describes the minimum certainty of the correct class to be accepted. Example: For a threshold of 0.75, an image is considered as correctly classified, if the correct class is (1) given the highest probability of all classes and (2) this probability is 0.75 or higher. Reading example for Figure **(A)**: At a threshold of 0.75, the accuracy is ≈ 0.88, however $\approx 18\%$ of the images cannot be classified, since the certainty is too high (Dropout Share ≈ 0.18).

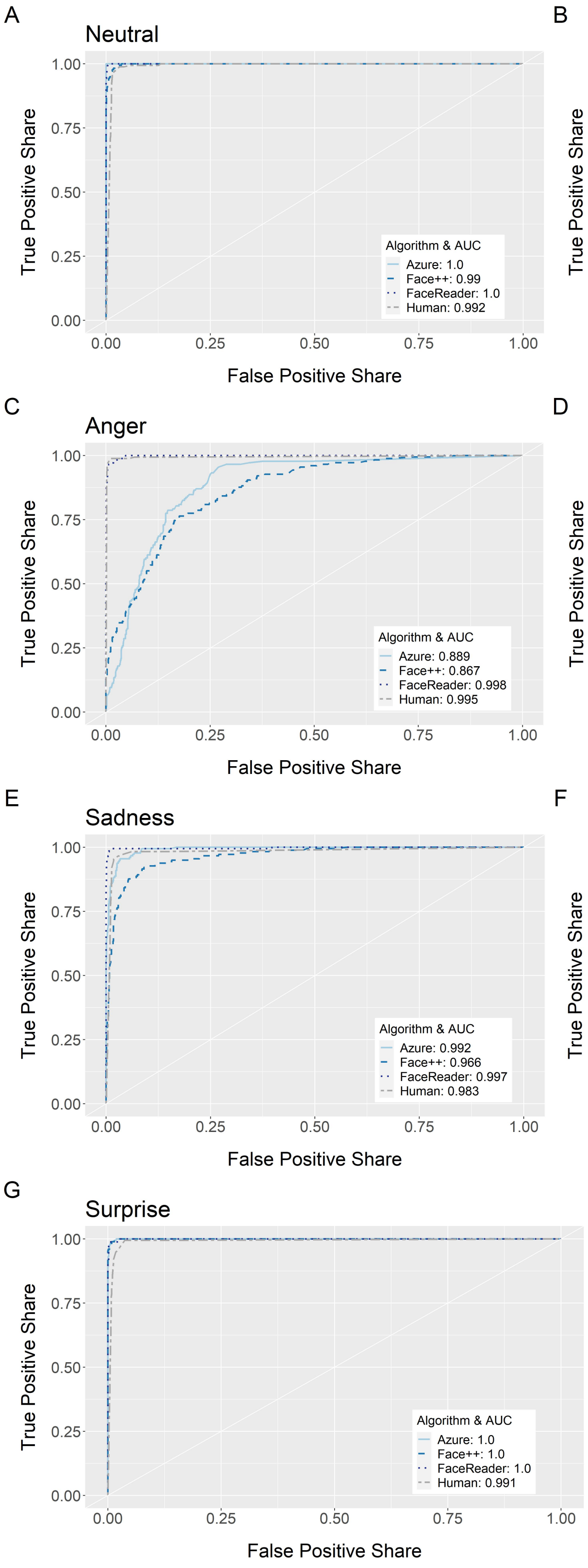

Figure S2. This Figure depicts ROC curves and the AUCs in the standardized data for each category: **(A)** for neutral, **(B)** for joy, **(C)** for anger, **(D)** for disgust, **(E)** for Sadness, **(F)** for fear, and **(G)** for surprise.

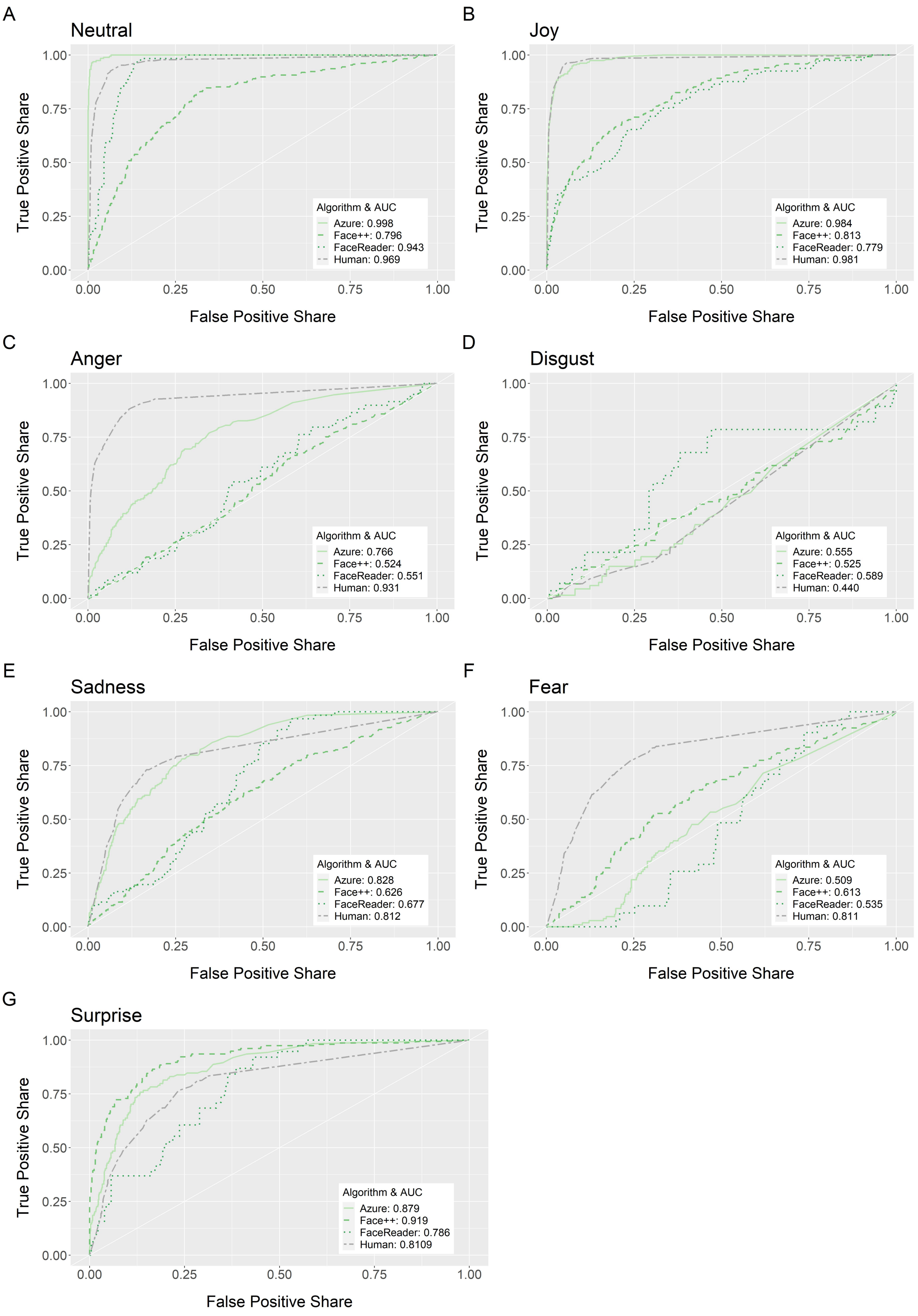

Figure S3. This Figure depicts ROC curves and the AUCs in the non-standardized data for each category: **(A)** for neutral, **(B)** for joy, **(C)** for anger, **(D)** for disgust, **(E)** for sadness, **(F)** for fear, and **(G)** for surprise.

1.2 Tables

Table S1. Pairwise X^2 Comparisons of Drop Out Rates

Algorithm 1	Algorithm 2	Data	Drop Out 1	Drop Out 2	X^2	p
Face++	Azure	Stand.	0.0	0.0	–	–
Face++	FaceReader	Stand.	0.0	0.88	9.13	.003
FaceReader	Azure	Stand.	0.88	0.0	9.13	.003
Face++	Azure	Non-Stand.	0.5	20.3	289.99	<.001
Face++	FaceReader	Non-Stand.	0.5	74.2	1606	<.001
FaceReader	Azure	Non-Stand.	74.2	20.3	804.89	<.001

Note: Degrees of Freedom are = 1 for all tests. ‘Stand.’ describes the standardized data. ‘Non-Stand.’ describes the non-standardized data. Drop Out rates are given in %.

Table S2. Pairwise X^2 Comparisons of Accuracies

Algorithm 1	Algorithm 2	Data	Accuracy 1	Accuracy 2	X^2	p
Face++	Azure	Stand.	0.786	0.810	2.09	.148
Face++	FaceReader	Stand.	0.786	0.967	184.4	< .001
FaceReader	Azure	Stand.	0.967	0.810	152.10	< .001
Face++	Azure	Non-Stand.	0.322	0.571	153.43	< .001
Face++	FaceReader	Non-Stand.	0.322	0.310	0.15	.700
FaceReader	Azure	Non-Stand.	0.310	0.571	72.65	< .001
Face++	Azure	Non-St.-Sub.	0.316	0.577	46.45	< .001
Face++	FaceReader	Non-St.-Sub.	0.316	0.316	0.00	1.00
FaceReader	Azure	Non-St.-Sub.	0.316	0.577	46.45	< .001

Note: Degrees of Freedom are = 1 for all tests. ‘Stand.’ describes the standardized data. ‘Non-Stand.’ describes non-standardized data. ‘Non-St.-Sub.’ describes the subset of non-standardized images recognized by all algorithms. Values for accuracies are rounded.

Table S3. Share of Correctly Identified Images

	Azure		Face++		FaceReader	
	Stand.	Non-Stand.	Stand.	Non-Stand.	Stand.	Non-Stand.
Neutral	1.0	0.78	0.94	0.40	0.99	0.35
Joy	1.0	0.76	0.99	0.47	1.0	0.18
Anger	0.51	0.29	0.49	0.15	0.96	0.05
Disgust	0.85	0.08	0.89	0.16	0.97	0.06
Sadness	0.88	0.36	0.81	0.18	0.98	0.07
Fear	0.46	0.02	0.40	0.18	0.88	0.01
Surprise	0.98	0.46	0.97	0.68	0.98	0.17
Total	0.81	0.45	0.79	0.32	0.96	0.14

Note: ‘Stand.’ describes the standardized data. ‘Non-Stand.’ describes non-standardized data.

Table S4. Sensitivity, Precision & Accuracy for the Non-Standardized Data Subset Recognized by all Algorithms

	Azure		Face++		FaceReader	
	Sens	Prec	Sens	Prec	Sens	Prec
Neutral	0.93	0.38	0.38	0.37	0.68	0.21
Joy	0.93	0.85	0.51	0.76	0.42	0.92
Anger	0.32	0.82	0.11	0.38	0.14	0.47
Disgust	0.12	0.33	0.12	0.15	0.15	0.17
Sadness	0.49	0.76	0.15	0.31	0.17	0.32
Fear	0.00	0.00	0.07	0.06	0.00	0.00
Surprise	0.53	0.47	0.74	0.19	0.35	0.32
Average	0.47	0.52	0.3	0.32	0.27	0.35
Accuracy	0.58		0.32		0.32	

Note: The category distribution over the subset is as follows: Neutral: 60, Happy: 81, Angry: 57, Disgust: 26, Surprise: 34, Sad: 59, Fear: 28.

Table S5. Pairwise Bootstrapped Comparisons of AUCs

Category	Algorithm 1	Algorithm 2	Data	AUC 1	AUC 2	D	p
All	Face++	Azure	Stand.	0.964	0.975	−4.45	<0.001
All	Face++	FaceReader	Stand.	0.964	0.998	−12.23	<0.001
All	FaceReader	Azure	Stand.	0.998	0.975	9.92	<0.001
All	Human	Azure	Stand.	0.982	0.975	2.10	0.035
All	Human	Face++	Stand.	0.982	0.964	5.04	<0.001
All	Human	FaceReader	Stand.	0.982	0.998	−6.95	<0.001
All	Face++	Azure	Non-Stand.	0.698	0.858	−16.336	<0.001
All	Face++	FaceReader	Non-Stand.	0.698	0.712	−0.92	0.360
All	FaceReader	Azure	Non-Stand.	0.712	0.858	−9.44	<0.001
All	Human	Azure	Non-Stand.	0.874	0.858	1.90	0.058
All	Human	Face++	Non-Stand.	0.874	0.698	18.48	<0.001
All	Human	FaceReader	Non-Stand.	0.874	0.712	10.63	<0.001
All	Face++	Azure	Nat. Sub.	0.707	0.863	−10.10	<0.001
All	Face++	FaceReader	Nat. Sub.	0.707	0.717	−0.52	0.601
All	FaceReader	Azure	Nat. Sub.	0.717	0.863	−8.11	<0.001
Neutral	Face++	Azure	Stand.	0.999	1.0	−2.80	0.005
Neutral	Face++	FaceReader	Stand.	0.999	1.0	−2.43	0.015
Neutral	FaceReader	Azure	Stand.	1.0	1.0	−1.54	0.124
Neutral	Human	Azure	Stand.	0.992	1.0	−3.89	<0.001
Neutral	Human	Face++	Stand.	0.992	0.999	−3.27	0.001
Neutral	Human	FaceReader	Protot	0.992	1.0	−3.70	<0.001
Joy	Face++	Azure	Stand.	1.0	1.0	−1.06	0.288
Joy	Face++	FaceReader	Stand.	1.0	1.0	−1.01	0.312
Joy	FaceReader	Azure	Stand.	1.0	1.0	0	1

Table continues on the next page.

Table S5. Pairwise Bootstrapped Comparisons of AUCs *(continued)*

Joy	Human	Azure	Stand.	0.997	1.0	−2.28	0.023
Joy	Human	Face++	Stand.	0.997	1.0	−2.22	0.027
Joy	Human	FaceReader	Protot	0.997	1.0	−2.26	0.024
Anger	Face++	Azure	Stand.	0.867	0.889	−1.68	0.093
Anger	Face++	FaceReader	Stand.	0.867	0.998	−9.94	<0.001
Anger	FaceReader	Azure	Stand.	0.998	0.889	9.90	<0.001
Anger	Human	Azure	Stand.	0.995	0.889	8.99	<0.001
Anger	Human	Face++	Stand.	0.995	0.867	9.49	<0.001
Anger	Human	FaceReader	Stand.	0.995	0.998	−0.93	0.353
Disgust	Face++	Azure	Stand.	0.994	0.971	3.68	<0.001
Disgust	Face++	FaceReader	Stand.	0.994	0.999	−2.98	0.003
Disgust	FaceReader	Azure	Stand.	0.999	0.971	4.19	<0.001
Disgust	Human	Azure	Stand.	0.979	0.971	0.76	0.45
Disgust	Human	Face++	Stand.	0.979	0.994	−2.28	0.023
Disgust	Human	FaceReader	Protot	0.979	0.999	−3.09	0.002
Sadness	Face++	Azure	Stand.	0.966	0.992	−4.60	<0.001
Sadness	Face++	FaceReader	Stand.	0.966	0.997	−4.62	<0.001
Sadness	FaceReader	Azure	Stand.	0.997	0.992	1.75	0.079
Sadness	Human	Azure	Stand.	0.983	0.992	−1.63	0.104
Sadness	Human	Face++	Stand.	0.983	0.966	1.92	0.055
Sadness	Human	FaceReader	Protot	0.983	0.997	−2.30	0.021
Fear	Face++	Azure	Stand.	0.767	0.852	−5.22	<0.001
Fear	Face++	FaceReader	Stand.	0.767	0.984	−10.89	<0.001
Fear	FaceReader	Azure	Stand.	0.984	0.852	8.79	<0.001
Fear	Human	Azure	Stand.	0.908	0.852	2.99	0.003
Fear	Human	Face++	Stand.	0.908	0.767	5.99	<0.001
Fear	Human	FaceReader	Protot	0.908	0.984	−5.94	<0.001
Surprise	Face++	Azure	Stand.	1.0	1.0	0.49	0.627
Surprise	Face++	FaceReader	Stand.	1.0	1.0	0.29	0.772
Surprise	FaceReader	Azure	Stand.	1.0	1.0	−0.09	0.931
Surprise	Human	Azure	Stand.	0.991	1.0	−2.51	0.012
Surprise	Human	Face++	Stand.	0.991	1.0	−2.48	0.013
Surprise	Human	FaceReader	Protot	0.991	1.0	−2.51	0.012
Neutral	Face++	Azure	Non-Stand.	0.796	0.998	−11.76	<0.001
Neutral	Face++	FaceReader	Non-Stand.	0.796	0.943	−7.81	<0.001
Neutral	FaceReader	Azure	Non-Stand.	0.943	0.998	−5.06	<0.001
Neutral	Human	Azure	Non-Stand.	0.969	0.998	−4.34	<0.001
Neutral	Human	Face++	Non-Stand.	0.969	0.796	10.33	<0.001
Neutral	Human	FaceReader	Non-Stand.	0.969	0.943	2.09	0.037
Joy	Face++	Azure	Non-Stand.	0.813	0.984	−12.07	<0.001
Joy	Face++	FaceReader	Non-Stand.	0.813	0.779	1.05	0.291
Joy	FaceReader	Azure	Non-Stand.	0.779	0.984	−7.29	<0.001

Table continues on the next page.

Table S5. Pairwise Bootstrapped Comparisons of AUCs *(continued)*

Joy	Human	Azure	Non-Stand.	0.981	0.984	−0.952	<0.554
Joy	Human	Face++	Non-Stand.	0.981	0.813	11.21	<0.001
Joy	Human	FaceReader	Non-Stand.	0.981	0.779	7.21	<0.001
Anger	Face++	Azure	Non-Stand.	0.524	0.766	−9.15	<0.001
Anger	Face++	FaceReader	Non-Stand.	0.524	0.551	−0.62	0.536
Anger	FaceReader	Azure	Non-Stand.	0.551	0.766	−5.20	<0.001
Anger	Human	Azure	Non-Stand.	0.931	0.766	7.95	<0.001
Anger	Human	Face++	Non-Stand.	0.931	0.524	18.79	<0.001
Anger	Human	FaceReader	Non-Stand.	0.931	0.551	9.70	<0.001
Disgust	Face++	Azure	Non-Stand.	0.525	0.555	−0.60	0.550
Disgust	Face++	FaceReader	Non-Stand.	0.525	0.589	−0.89	0.373
Disgust	FaceReader	Azure	Non-Stand.	0.589	0.555	0.49	0.625
Disgust	Human	Azure	Non-Stand.	0.440	0.555	−2.65	0.008
Disgust	Human	Face++	Non-Stand.	0.931	0.525	−2.02	0.043
Disgust	Human	FaceReader	Non-Stand.	0.931	0.589	−2.20	0.278
Sadness	Face++	Azure	Non-Stand.	0.626	0.828	−8.79	<0.001
Sadness	Face++	FaceReader	Non-Stand.	0.626	0.677	−2.09	0.036
Sadness	FaceReader	Azure	Non-Stand.	0.677	0.828	−4.64	<0.001
Sadness	Human	Azure	Non-Stand.	0.812	0.828	−0.76	0.448
Sadness	Human	Face++	Non-Stand.	0.812	0.626	8.41	<0.001
Sadness	Human	FaceReader	Non-Stand.	0.812	0.677	4.12	<0.001
Fear	Face++	Azure	Non-Stand.	0.613	0.509	2.85	0.004
Fear	Face++	FaceReader	Non-Stand.	0.613	0.535	1.54	0.122
Fear	FaceReader	Azure	Non-Stand.	0.535	0.509	0.52	0.600
Fear	Human	Azure	Non-Stand.	0.811	0.509	9.03	≪0.001
Fear	Human	Face++	Non-Stand.	0.811	0.613	6.24	<0.001
Fear	Human	FaceReader	Non-Stand.	0.811	0.535	5.81	<0.001
Surprise	Face++	Azure	Non-Stand.	0.919	0.879	1.92	0.055
Surprise	Face++	FaceReader	Non-Stand.	0.919	0.786	3.86	<0.001
Surprise	FaceReader	Azure	Non-Stand.	0.786	0.879	−2.57	0.101
Surprise	Human	Azure	Non-Stand.	0.809	0.879	−2.77	0.006
Surprise	Human	Face++	Non-Stand.	0.809	0.919	−4.68	<0.001
Surprise	Human	FaceReader	Non-Stand.	0.809	0.786	0.62	0.535

Note: *Stand.* is short for standardized data. *Non-Stand.* is short for non-standardized data. *Nat. Sub.* is short for the subset of non-standardized images recognized by all algorithms. Values for AUCs, D and p are rounded.
